# Dietary Fiber-Rich *Spartina anglica* Improves Intestinal Health and Antioxidant Capacity of Zhedong White Geese

**DOI:** 10.3390/antiox14010087

**Published:** 2025-01-13

**Authors:** Xiao Zhou, Li Wang, Jiuli Dai, Huiyan Jia, Kai Shi, Jian Zhao, Shufang Chen

**Affiliations:** Livestock and Poultry Research Institute, Ningbo Academy of Agriculture Sciences, Ningbo 315000, China; xiaozhou_2023@163.com (X.Z.); q15928806587@163.com (L.W.); 13858357201@163.com (J.D.); jhynku@163.com (H.J.); shikai2024@126.com (K.S.)

**Keywords:** *Spartina anglica*, goose, microbial community, antioxidant capacity, intestinal health

## Abstract

*Spartina anglica* (SA), a plant rich in dietary fiber, has demonstrated considerable potential for enhancing gut health and antioxidant capacity in animals. This study investigates the integration of SA as a novel dietary ingredient for Zhedong white geese, with a specific focus on evaluating its effects on growth performance, nutrient digestibility, antioxidant capacity, intestinal health, and cecal microbiota composition. A total of 360 1-day-old Zhedong white geese with an average weight of 114.94 ± 0.81 g were randomly allocated to 4 dietary treatments, with 6 replicates per treatment and 15 geese per pen. The 4 dietary treatments included different SA supplement levels: a control group receiving a basal diet (CON), and three experimental groups supplemented with 3% SA (SA3), 6% SA (SA6), and 12% SA (SA12). Supplementation with 6% SA significantly enhanced the final body weight, average daily gain, and feed conversion ratio (FCR) compared to the CON group (*p* < 0.05). In contrast, the SA12 group exhibited reduced digestibility of crude protein and ether extract, relative to the SA3 and SA6 groups (*p* < 0.05). The highest antioxidant capacity was observed in the SA6 and SA12 groups, while the lowest was recorded in the CON group. SA supplementation did not significantly influence serum biochemical parameters or organ indices but increased cecum length (*p* < 0.05). Notably, SA supplementation markedly improved intestinal morphology, although excessive levels appeared to compromise these benefits. Additionally, SA supplementation significantly enhanced the richness and diversity of cecal microbiota and increased short-chain fatty acid concentrations. In conclusion, SA at an optimal supplementation level of 6% may be effectively utilized in Zhedong white geese diets to improve growth performance, gut health, and antioxidant capacity.

## 1. Introduction

Poultry production faces increasing challenges in maintaining optimal growth performance and health under intensive farming conditions. Among these challenges, oxidative stress and gut health have drawn particular attention, as they not only influence animal welfare but also affect feed efficiency, meat quality, and overall productivity in the poultry industry [1,2]. In recent years, research has focused on the use of functional feed additives, including those rich in bioactive components such as antioxidants and dietary fibers, to support both gut integrity and systemic antioxidant status in birds [3].

*Spartina anglica* (SA), a halophyte plant widely found in coastal regions, is recognized for its high fiber content and robust adaptability to challenging environmental conditions [4]. Previous studies have shown that dietary fiber, when properly included in poultry diets, can confer multiple benefits such as improving intestinal health, modulating the gut microbiota, and enhancing nutrient utilization [5]. Moreover, fibers may serve as prebiotics, supporting beneficial microbial populations and increasing the production of short-chain fatty acids (SCFAs), which have been linked to improved intestinal integrity and overall health in birds [6]. However, the inclusion of high-fiber feedstuffs must be carefully regulated to avoid potential reductions in nutrient digestibility and growth performance.

Research on Spartina species as a feed ingredient in poultry diets remains limited, despite initial findings suggesting its potential to enhance antioxidant capacity and gut health in animals [7]. In geese, which have distinctive digestive physiology characterized by a well-developed cecum, novel fiber sources may offer unique advantages by promoting a beneficial microbial ecosystem and bolstering natural defenses against oxidative stress [8]. Notably, the Zhedong white goose is a valuable native breed in China, recognized for its adaptability and economic importance [9].

Given this background, the current study was designed to evaluate the impact of SA supplementation in diets for Zhedong white geese, with a particular focus on growth performance, nutrient digestibility, antioxidant capacity, intestinal morphology, and the composition of cecal microbiota. By systematically examining the optimal supplementation concentration of SA (3%, 6%, and 12%), we aimed to identify an optimal dosage that balances the benefits of fiber-rich feed ingredients with the maintenance of performance parameters.

## 2. Materials and Methods

### 2.1. Experimental Design and Diets

All procedures used in animal experiments in this study were approved by the Ethics Committee for Animal Experiments of Ningbo Academy of Agriculture Sciences (Ningbo, China). A total of 360 1-day-old Zhedong white geese (with an initial body weight of 114.94 ± 0.81 g) were purchased from Zhejiang Jiuxu White Geese Company (Ningbo, China) and fed until 70 days. All geese were randomly allotted into 4 treatments with 6 replicates per treatment and 15 geese per pen, and the treatments were as follows: a basal diet (CON), a basal diet + 3% SA (SA3), a basal diet + 6% SA (SA6), and 12% SA (SA12). The SA used in this experiment was granular and was crushed through a 2 mm screen prior to usage. The SA was purchased by Xiangshan Liangxin Co., Ltd. (Shanghai, China), and the nutrient levels are shown in Table 1. Geese were allowed ad libitum access to feed and water during the whole experimental period. The experiment diets were formulated to meet nutrient levels recommended by the National Research Council (National Research Council, 1994) for geese [10]. The composition and nutrient levels (as-fed basis) of the CON diet are shown in Table 2. In the SA-supplemented groups, equal proportions of corn were replaced with varying doses of SA, while energy and amino acid levels were adjusted using soybean oil and industrial amino acids. The growth performance, nutrient digestibility, antioxidant capacity, intestinal morphology, and the composition of cecal microbiota were measured to evaluate the impact of SA supplementation in diets for Zhedong white geese and find the optimal supplementation.

### 2.2. Sample Collection

On day 1 and day 42, approximately 1 kg of representative feed samples were randomly collected from various bag locations using a bag trier for analysis. From day 67 to day 70, excreta were collected twice daily using collection plates placed under each pen, with approximately 50 g per collection. Feathers and feed residues were removed before storage at −20 °C. After the collection period, excreta samples were thawed, thoroughly mixed, dried at 65 °C for 72 h, and ground to pass through a 1 mm screen.

On day 70, one bird per pen (15 birds per treatment) with a body weight close to the pen’s average was euthanized by electrical stunning and carotid artery bleeding. Blood samples (10 mL) were collected from the left atrium using anticoagulant vacuum tubes. Tissue samples (1 cm) were taken from the midsections of the jejunum and ileum, rinsed with phosphate-buffered saline, and fixed in 10% formaldehyde. Cecum digesta and liver samples (1 cm) were flash-frozen in liquid nitrogen and stored at −80 °C for further analysis.

### 2.3. Chemical Analysis

Samples of ingredients and diets were ground through a 1 mm screen and analyzed according to the Official Methods of Analysis of the Association of Official Agricultural Chemists (AOAC) [11], including dry matter (DM: AOAC, 2007; method 930.15), crude protein (CP: AOAC, 2007; method 976.05), and ash (AOAC, 2007; method 942.15). Crude fiber, neutral detergent fiber (NDF), and acid detergent fiber (ADF) were determined using a fiber analyzer (Ankom Technology, Macedon, NY, USA) following the method of Van Soest et al., with heat-stable α-amylase and sodium sulfite, and were expressed inclusive of residual ash [12]. Gross energy was measured using an automatic adiabatic oxygen bomb calorimeter (Parr 6300 Calorimeter, Moline, IL, USA). Acid-insoluble ash (AIA) concentrations in feed and excreta samples were determined by atomic absorption spectrometry (Z-5000; Hitachi, Tokyo, Japan) following the protocol described by García-Rico et al. [13].

### 2.4. Growth Performance and Nutrient Digestibility

On days 42 and 70, geese were fasted for 12 h, after which individual body weight and feed consumption were measured to calculate average daily gain (ADG), average daily feed intake (ADFI), and feed conversion ratio (FCR).

After the determination of DM, CP, ash, CF, NDF, ADF, and the Cr_2_O_3_ concentrations in feces and feeds was finished, the apparent total tract digestibility (ATTD) of nutrients was calculated according to Liu [14], and the function is as follows:

ATTD (%) = [1 − (content of Cr in diets × content of nutrients in excreta)/(content of Cr in excreta x content of nutrients in diets)] × 100%.

### 2.5. Serum Biochemical Parameters

Blood samples collected from the selected geese were analyzed to evaluate serum biochemical parameters [15]. The activity levels of alanine aminotransferase (ALT) and aspartate aminotransferase (AST), along with the concentrations of total protein (TP), albumin (ALB), globulin (GLOB), blood urea nitrogen (BUN), cholesterol (CHO), triglycerides (TG), high-density lipoprotein (HDL), and low-density lipoprotein (LDL), were measured using an automatic biochemical analyzer (AU680, Beckman Coulter., Tokyo, Japan).

### 2.6. Antioxidant Capacity

After being thawed at 4 °C, liver samples were homogenized in isotonic sodium chloride solution at a 1:9 (*w*/*v*) ratio at 4 °C, followed by centrifugation at 3000× *g* for 10 min. The supernatants of liver tissue and serum were collected to determine the total antioxidant capacity (T-AOC) and glutathione peroxidase (GSH-Px), superoxide dismutase (SOD), catalase (CAT), and malondialdehyde (MDA) levels [16]. Test kits were obtained from Nanjing Jiancheng Bioengineering Institute (Nanjing, China), and the assay followed the manufacturer’s instructions precisely. In detail, the procedure involved setting up blank, standard, and sample wells. For the standard wells, 50 µL of the standard sample was pipetted onto the enzyme-coated plate. In the sample wells, 40 µL of sample diluent and 10 µL of the sample were added, resulting in a final fivefold dilution. The plate was sealed with a film and incubated at 37 °C for 30 min. A 30-fold dilution of the concentrated washing solution with distilled water was prepared for subsequent washing steps. Following the washing steps, 50 µL of enzyme-labeled reagent was added to each well, except for the blank wells, and the plate was incubated again. Next, 50 µL each of color reagent A and color reagent B were sequentially added, and the plate was gently shaken before incubating at 37 °C for 10 min. The reaction was terminated by adding 50 µL of termination solution to each well. Optical density (OD) values were measured at 450 nm using a multifunctional microplate reader (SpectraMax i3X A1705, Molecular Devices, LLC, San Jose, CA, USA), with blank wells set to zero. All measurements were completed within 15 min after the termination solution was added.

### 2.7. Organ Index and Intestinal Development

On day 70, prior to slaughter, each chosen goose was weighed. Post-slaughter, organs including the pancreas, kidneys, heart, liver, gallbladder, spleen, lungs, uropygial gland, thymus gland, bursa of fabricius, gizzard, and glandular stomach were carefully isolated from all connective tissue and fat and then weighed. The lengths of the duodenum, jejunum, ileum, and cecum were quantified using an electronic scale. The organ index was determined by dividing the weight of each organ by the corresponding body weight of the goose [15].

### 2.8. Intestinal Morphology

The jejunum and ileum tissues were dehydrated using a graded ethanol–xylene series and embedded in paraffin. Paraffin sections with a thickness of 5 mm were prepared, mounted on slides, stained with hematoxylin and eosin, and examined under a digital camera microscope (NE 900, Ningbo Yong Xin Optics Co., Ltd., Ningbo, China). Morphological images were captured, and villus height (VH) and crypt depth (CD) were measured using the NE 900 digital image analysis system. The villus height-to-crypt depth ratio (VH/CD) was determined by dividing VH by CD.

### 2.9. Cecum Microbiota Analysis

Total genomic DNA was isolated from cecum digesta using the NEB Next^®^ Ultra™ DNA Library Prep Kit, in accordance with the manufacturer’s guidelines. The final concentration and purity of DNA were ascertained using a NanoDrop 2000 UV-Vis spectrophotometer (Thermo Scientific, Wilmington, DE, USA). Furthermore, the quality of DNA was verified via 2% agarose gel electrophoresis. V3-V4 regions of bacterial 16S ribosomal RNA genes were amplified through polymerase chain reaction (PCR), utilizing primers 338F (5′-ACTCCTACGGGAGGCAGCAG-3′) and 806R (5′-GGACTACHVGGGTWTCTAAT-3′). The established amplification procedure encompassed denaturation at 98 °C for 1 min, followed by 30 cycles each of 98 °C for 10 s, annealing for 30 s at 50 °C, and elongation for 20 s at 72 °C. This was concluded by an extension stage for 5 min at 72 °C. Amplicon quality was checked using gel electrophoresis. The purified amplicons were pooled homogenously, and paired-end sequencing (2 × 300) was performed on an Illumina MiSeq platform following standard protocols. Raw fastq files underwent demultiplexing and quality filtration using QIIME (version 1.17). Furthermore, operational taxonomic units (OTUs) were recognized with a similarity threshold of 0.97 using UPARSE. The taxonomy of each 16S rRNA gene sequence was evaluated using the RDP Classifier algorithm (http://rdp.cme.msu.edu/ (accessed on 18 November 2024)) against the Silva (SSU128) 16S rRNA database employing a confidence threshold of 80% [17].

### 2.10. Short-Chain Fatty Acids in Cecum

The content of short-chain fatty acids in the cecum digesta was analyzed for evaluating the fermentation among three groups, based on the methods of He et al. [18]. Cecum digesta samples were thawed on ice and thoroughly mixed. From this mixed solution, 1.0 g samples were suspended in 8 mL of deionized water and then subjected to a 20 min ultrasonic irradiation, followed by centrifugation at 12,000× *g* for 10 min. The supernatant was subsequently diluted 50-fold and passed through a 0.22 mm filter. The resultant sample solution underwent analysis with a high-performance ion chromatograph ICS-3000 (Dionex, Sunnyvale, CA, USA). Concentrations of short-chain fatty acids (SCFAs), inclusive of acetic acid, propanoic acid, butyric acid, valeric acid, and isovaleric acid, were quantified and expressed as mg/kg of digesta.

### 2.11. Statistical Analysis

Growth performance, nutrient digestibility, antioxidant capacity, serum biochemical parameters, organ index, intestinal length, intestinal morphology, and SCFA concentrations were analyzed using one-way ANOVA in SPSS (version 22.0; SPSS Inc., Armonk, NY, USA), followed by Tukey’s multiple range test. Linear and quadratic effects of SA supplementation were assessed using orthogonal polynomials. Results presented in the tables are expressed as means with pooled standard error of the mean (SEM). A *p*-value < 0.05 was considered statistically significant.

## 3. Results

### 3.1. Growth Performance

Growth performance results are shown in Table 3, with SA6 demonstrating the most favorable results. On day 70, geese in the CON and SA12 groups had significantly lower final body weight and average daily gain (ADG) than those in the SA3 and SA6 groups (*p* < 0.05). The SA12 group also exhibited a lower average daily feed intake (ADFI) compared to the other three groups (*p* < 0.05). Additionally, the SA6 group had the lowest feed conversion ratio (FCR) of the four groups, significantly lower than those in the CON and SA12 groups (*p* < 0.05). Quadratic effects were observed on final body weight, ADG, ADFI, and FCR (*p* < 0.05), with an optimal turning point at the 6% SA level, except for ADFI.

### 3.2. Nutrient Digestibility

The ATTD of nutrients is presented in Table 4, revealing significant differences between the SA6 and SA12 groups. In crude protein digestibility, the SA3 and SA6 groups had significantly higher values than the SA12 group (*p* < 0.05). For ether extract, the SA12 group displayed lower digestibility than the other three groups (*p* < 0.05). No significant differences were found for dry matter and organic matter digestibility (*p* = 0.07 and *p* = 0.21, respectively). Furthermore, significant linear and quadratic effects were identified for the digestibility of crude protein and ether extract (*p* < 0.05), and the optimal nutrient digestibility was observed at the SA6 supplementation level.

### 3.3. Serum Biochemical Parameters

Table 5 provides the results of the serum biochemical analysis, including measurements of ALT, AST, TP, ALB, GLOB, BUN, CHO, TG, HDL, and LDL. No differences were observed among the four groups and neither linear nor quadratic effects were detected (*p* > 0.05).

### 3.4. Antioxidant Capacity

The activities of antioxidant enzymes, including SOD, CAT, T-AOC, GSH-Px, and MDA concentrations, in the liver and serum of Zhedong white geese were analyzed, as shown in Table 6 and Table 7. SA supplementation significantly increased liver SOD, CAT, and T-AOC activities compared to the CON group (*p* < 0.01). Additionally, liver GSH-Px levels in the SA6 and SA12 groups were markedly higher than those in the CON group (*p* < 0.01). In contrast, no significant differences were observed in liver MDA concentrations or in serum SOD, CAT, GSH-Px, and MDA levels (*p* < 0.05). The only significant serum difference was an elevated T-AOC level in the SA6 group compared to the CON group (*p* < 0.05). Both linear and quadratic effects of SA supplementation were noted for liver SOD, CAT, T-AOC, and GSH-Px activities, with a linear effect also identified for T-AOC in serum (*p* = 0.03). The SA6 and SA12 groups demonstrated the highest antioxidant capacity in the liver and serum.

### 3.5. Organ Index

The effects of SA supplementation on organ indices are presented in Table 8. Analysis of these results revealed no statistically significant differences attributable to SA supplementation (*p* > 0.05).

### 3.6. Intestinal Development and Morphology

The effects of SA supplementation on intestinal development are presented in Table 9. No significant differences were observed in gizzard weight or in the lengths of the duodenum, jejunum, and ileum (*p* > 0.05). However, cecum length demonstrated a significant linear increase with SA supplementation, with the cecum length in the SA12 group being significantly greater than in the CON and SA3 groups (*p* < 0.05).

**Figure 1:**
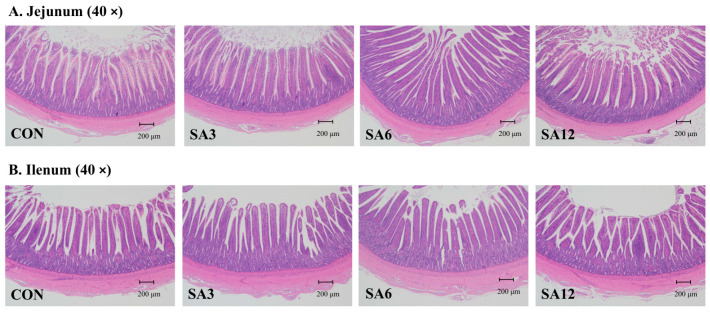
Intestinal morphology at 40× magnification: (**A**) jejunum and (**B**) ileum. The SA3, SA6, and SA12 groups were the control diets supplemented with 3%, 6%, and 12% SA, respectively.

Supplementation with 12% SA significantly increased crypt depth and reduced the villus height-to-crypt depth ratio (V/H) in the jejunum compared to the SA3 and SA6 groups (Table 10, *p* < 0.05). A similar effect was noted in the ileum, where the SA12 group showed the greatest crypt depth and lowest V/H ratio. Significant linear and quadratic effects were observed for both crypt depth and V/H ratio (*p* < 0.05). A 40× magnified field view from an electron microscope is presented in Figure 1.

### 3.7. Cecum Microbiota Analysis

**Figure 2:**
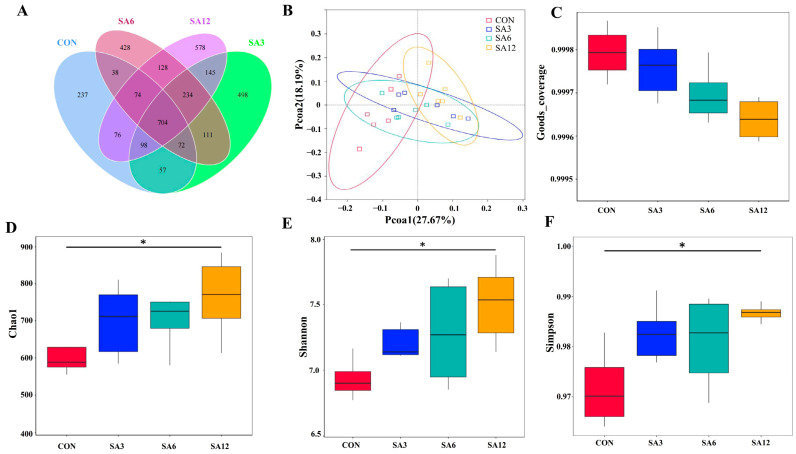
Effects of *Spartina anglica* (SA) supplementation on microbial diversity in the cecum of Zhedong white geese: (**A**) distribution of operational taxonomic units (OTUs) in the cecum; (**B**) principal coordinates analysis (PCoA) of beta diversity; (**C**–**F**) alpha diversity metrics of the cecum, including Good’s coverage, Chao1, Shannon, and Simpson indices. The statistical significance is indicated as * *p* < 0.05. The SA3, SA6, and SA12 groups were the control diets supplemented with 3%, 6%, and 12% SA, respectively.

Figure 2 presents indicators of cecum microbial diversity. Figure 2A shows a Venn diagram of the OTUs identified in each group, with a total of 3478 OTUs observed across all groups. Among these, 704 OTUs were shared, while unique OTUs were identified as follows: 237 in the CON group, 498 in SA3, 428 in SA6, and 578 in SA12, highlighting intergroup diversity. The Principal Coordinates Analysis (PCoA) plot in Figure 2B illustrates a distinct separation in microbial communities on the PCoA1 axis between the CON and SA12 groups, suggesting an alteration in microbial composition due to 12% SA supplementation. Good’s coverage values showed no significant difference across groups (Figure 2C). The SA12 group, compared to the CON group, exhibited significantly higher Chao1, Shannon, and Simpson diversity indices (Figure 2D–F, *p* < 0.05).

**Figure 3:**
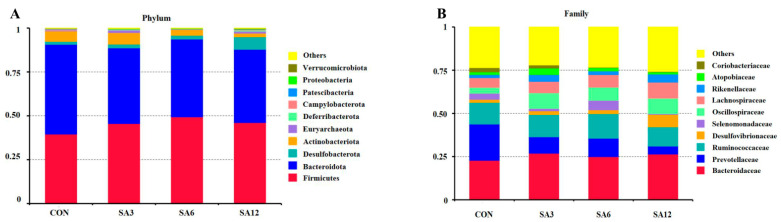
The distribution of cecal microbiota in the goose: (**A**) phylum level and (**B**) family level. *n* = 6 for each group. The SA3, SA6, and SA12 groups were the control diets supplemented with 3%, 6%, and 12% SA, respectively.

Figure 3 illustrates the relative abundance of cecal microbes at both the phylum and family levels. Firmicutes and Bacteroidetes were the dominant phyla. Compared to the CON group, SA-supplemented groups showed a reduction in the relative abundance of Bacteroidota. The most notable change was an increase in Desulfobacterota relative abundance when the diet included 12% SA (Figure 3A). At the family level, SA supplementation promoted the proliferation of Desulfovibrionaceae, Oscillospiraceae, Lachnospiraceae, and Rikenellaceae, while inhibiting the growth of Prevotellaceae in the geese’s cecum (Figure 3B).

**Figure 4:**
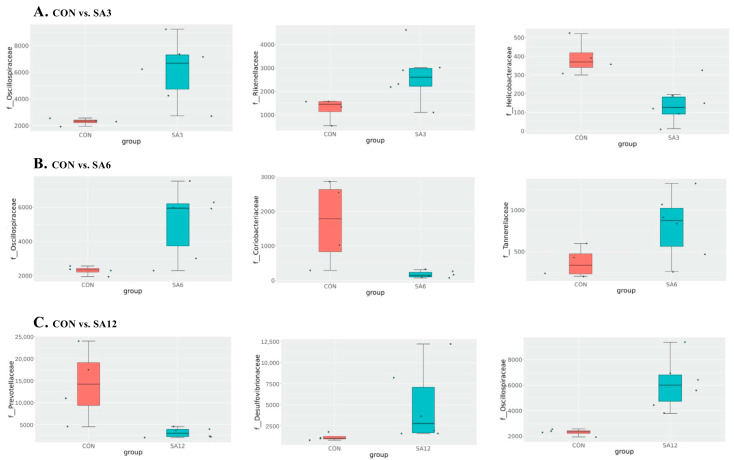
MetaStat analysis of the top three differential species at the family level: (**A**) CON vs. SA3, (**B**) CON vs. SA6, and (**C**) CON vs. SA12. *n* = 6 for each group. The SA3, SA6, and SA12 groups were the control diets supplemented with 3%, 6%, and 12% SA, respectively.

Figure 4 presents the intergroup differences in microbial composition. Oscillospiraceae levels were significantly increased in all groups with SA supplementation compared with the CON group. Compared to the CON group, the SA3 group showed an increase in Rikenellaceae and a decrease in Helicobacteraceae. In the SA6 group, Coriobacteriaceae levels were reduced while Tannerellaceae levels increased. In the SA12 group, Prevotellaceae was decreased, and Desulfovibrionaceae was elevated (Figure 4A–C).

Table 11 shows the concentrations of cecum SCFAs. The levels of acetic acid, propanoic acid, butyric acid, and isovaleric acid were significantly higher in the SA12 group than in the CON group (*p* < 0.05). Linear effects were observed for acetic acid, propanoic acid, butyric acid, and isovaleric acid concentrations in the cecum (*p* < 0.05), while no quadratic effects were detected for any SCFA (*p* > 0.05).

## 4. Discussion

### 4.1. SA Improves Small Intestine Morphology and Nutrient Digestibility

Given that SA contains 33.15% cellulose and 27.13% hemicellulose, both of which are major components of dietary fiber, over half of SA’s composition consists of dietary fiber [19]. The primary dietary modification from adding SA is an increase in dietary fiber. Compared to other monogastric animals, birds typically have a shorter and lighter gastrointestinal tract (GIT). While adapting to roughage, including herbaceous feed, dietary fiber significantly influences the intestinal development of Zhedong white geese. Villi, which are essential for nutrient absorption, consist of regions that include crypts and are replenished by stem cells [20]. Consequently, any reduction in VH reduces the surface area available for nutrient absorption and the secretion of digestive enzymes. Conversely, an increase in CD, indicative of active cell replacement, results in decreased absorption [21,22]. Thus, the VH/CD ratio has become an important indicator of GIT development and digestive capacity. In this study, SA supplementation improved intestinal morphology; however, an excessive level of SA (12%) had adverse effects. This finding is consistent with Jiménez-Moreno et al. [23], who reported that adding 2.5% pea hulls or sugar beet pulp improved the VH/CD ratio in broilers, whereas a 7.5% dietary inclusion led to villus shortening and increased mucus output. Similar results were observed by Koçer et al. [24], where adding 47 g of sunflower meal per kg of the diet increased the VH/CD ratio, which then decreased with 97 g of sunflower meal per kg in laying hens. This phenomenon may be attributed to the negative impact of excessive fiber intake, which can lead to disruption of intestinal villi due to fiber particle abrasion [19].

The small intestine is the principal organ responsible for nutrient digestion and absorption. Changes in digestibility closely follow the trends observed in small intestine morphology. Excessive fiber consumption can erode the villi of the small intestine, leading to a decrease in VH and consequently reducing the surface area for nutrient digestion. The alterations in the morphology of the jejunum and ileum due to SA supplementation are likely the main contributors to the differences in nutrient digestibility observed in Zhedong white geese. Additionally, the solubility and water-holding capacity of fiber can significantly affect nutrient utilization. A diet high in fiber content may increase viscosity, slowing the diffusion rate of endogenous enzymes into the digesta, and potentially reducing nutrient digestion [25].

### 4.2. SA Increases Cecum Length and Improves Gut Microbial Structure

SA supplementation influenced the relative length of the cecum. This is partially consistent with the findings of Li et al. [26], who indicated that feeding geese β-glucan influenced not only the cecum but also the duodenum, ileum, and colon. The cecum is the primary site of dietary fiber fermentation. An increase in dietary fiber intake leads to digestive bulk and physical distension of the intestinal walls, resulting in an increase in size. Despite evidence indicating a link between fiber type and quantity with organ development [27], our results revealed that SA supplementation had no effect on the development of organs other than the GIT. This aligns with Li et al. [28], who reported that diverse dietary fibers (2.5–6.1%) did not influence the relative weight of the liver, spleen, bursa, thymus, etc.

The poultry microbiota comprises a myriad of bacteria, including beneficial species involved in critical biological processes, such as the production of digestive enzymes, regulation of leukocyte activity, and amino acid metabolism. These beneficial species contrast with harmful bacteria that can invade the GIT and disrupt normal functioning by producing toxins [29]. A balanced and stable microbiota in the GIT is crucial for maintaining intestinal health. The Shannon and Simpson indices are routinely employed to evaluate bacterial diversity, while Chao1 measures the richness of the gut microbiome. A diverse gut microbiome enhances resistance to colonization by pathogens and reduces the risk of GIT diseases [30,31]. In this study, a notable increase in the diversity and abundance of cecum microbiota was observed in the SA12 group. Li et al. [32] reported similar findings, showing that a reduction in dietary fiber levels diminished the Shannon and Chao1 indices of cecum microbiota in geese on day 70. SCFAs are the main metabolites produced by gut microbiota during dietary fiber fermentation in the cecum and are closely associated with the abundance of gut microbiota [33,34,35]. Multiple studies have documented that SCFA levels in the cecum increase with higher levels of dietary fiber [33,36]. The heightened SCFAs in SA supplementation groups suggest that SA supplementation could improve the diversity of cecum microbiota in Zhedong white geese.

Spartina anglica supplementation triggered changes in the cecum’s length, which in turn influenced microbial composition. In the cecum of Zhedong white geese, the dominant bacteria were Bacteroidetes, Firmicutes, and the Desulfobacterota phylum. This contradicts the findings of Li et al. [32], who identified Proteobacteria as the third most common bacteria in geese. Desulfobacterota, a sulfate-reducing bacteria, can inhibit enzymes involved in nicotinamide adenine dinucleotide recycling and ultimately remove hydrogen, which restricts SCFA production [37]. This may also explain why the SA12 group had the highest SCFA levels. Regardless of the proportion of SA added, the greatest effect on microorganisms at the genus level was an increase in Oscillospiraceae. Oscillospiraceae represents important producers of SCFAs in the gut, particularly butyric acid, which not only provides energy to the intestinal epithelium and enhances intestinal barrier function but also possesses anti-inflammatory properties that help reduce inflammatory responses and improve intestinal immune function [38]. The production of SCFAs reduces the pH level of the cecum, creating an acidic environment that inhibits harmful bacteria and promotes the growth of beneficial bacteria, thus maintaining a healthy microbial community. One noticeable change induced by 12% SA supplementation was the reduction of Prevotellaceae at the family level. Many studies have identified the degradation of plant-derived polysaccharides as an energy source for Prevotellaceae [39,40]. Although SA contains plant polysaccharides, its significant impact is an increase in insoluble fiber. This is corroborated by our findings and by Gálvez et al. [41], who observed stark reductions in the abundance of murine Prevotellaceae when replacing a diet rich in plant-derived polysaccharides with a diet containing starch and indigestible cellulose as fiber. Overall, SA supplementation altered the cecum microbiota and may influence pro-inflammatory cytokines, intestinal permeability, and gut barrier function by modulating the corresponding microbiota [42,43].

### 4.3. SA Improves Antioxidant Capacity and Growth Performance

Under normal physiological conditions, organisms cyclically produce reactive oxygen species (ROS) and eliminate them through both enzymatic and non-enzymatic antioxidant systems, maintaining dynamic equilibrium. The enzymatic antioxidant system produces enzymes such as SOD, CAT, and GSH-Px, which convert ROS into H_2_O and O_2_, thereby protecting the body from oxidative damage [44]. Total antioxidant capacity reflects the effectiveness of an animal’s non-enzymatic antioxidant defense system [45]. As the level of SA supplementation increases, the antioxidant capacity of Zhedong white geese improves, peaking at a 6% supplementation level, but antioxidant performance remains essentially unchanged at the 12% SA supplementation level. Although research on the use of SA in animal feed is limited, it is widely accepted that plant-based feed ingredients enhance antioxidant capabilities. Liu et al. [14] demonstrated that supplementing broiler diets with 80 mg/kg of natural capsaicin extract increased antioxidant capacity. Similarly, the inclusion of Artemisia annua in feed has been shown to reduce MDA levels and upregulate antioxidant enzyme expression [46]. Plant-derived fibers are typically rich in polyphenolic compounds, which can inhibit free radical formation and reduce their activity [47,48]. Additionally, an increase in SCFAs, particularly butyrate, provides energy to intestinal epithelial cells, maintaining their integrity and health. This effectively prevents toxins and pathogens from entering the bloodstream, reducing oxidative stress sources. SCFAs also promote the expression of antioxidant enzymes via cellular signaling pathways, such as the Nrf2 pathway, thereby enhancing overall antioxidant capacity. However, compared to the SA6 group, the SA12 group shows no additional improvement in antioxidant performance. This phenomenon aligns with previous findings that polyphenolic compounds can exhibit antioxidant effects at low doses but pro-oxidant effects at high doses [49,50]. Furthermore, a 12% SA supplementation may cause intestinal villus abrasion, potentially offsetting the antioxidant benefits provided by polymeric polyphenols and other compounds.

Serum biochemical parameters are critical indicators of organ health and metabolic function [51]. ALT, AST, and TP levels reflect liver tissue and cell permeability, while BUN indicates the state of amino acid metabolism [52]. Our study found no significant effect of SA supplementation on the serum biochemical parameters of geese at any supplementation level. This suggests that dietary SA does not adversely affect liver or kidney function or related metabolic processes.

Growth performance is an integrated trait influenced by multiple factors, warranting its discussion toward the end. Studies have reported conflicting findings regarding the impact of dietary crude fiber on ADFI in poultry. While some studies [37,53] found no significant effects of fiber levels on feed intake, others, including Li et al. [26] and our research, observed an influence of dietary fiber on feed consumption. These discrepancies may stem from differences in the composition of soluble and insoluble fibers, which vary in water-holding and swelling capacities, influencing digesta viscosity and retention time in the GIT [19]. Supporting our findings, Zhang et al. [54] reported the highest ADG in geese fed a diet containing 6% crude fiber, among six graded levels (3%, 4.5%, 6%, 7.5%, 9%, and 10.5%).

Body growth largely depends on intestinal development, immune function, and nutrient absorption. Moderate SA supplementation (6%) significantly improves intestinal morphology and cecal microbiota composition. However, excessive supplementation (12%) impairs intestinal morphology and nutrient digestion, with the detrimental effects outweighing the beneficial ones. Additionally, antioxidant capacity, a key health indicator, significantly influences animal growth and metabolism, with SA6 being the most effective. Therefore, a 6% SA supplementation level is recommended for optimal performance. These findings hold promise for enhancing the nutritional strategies in goose production and contribute to the broader understanding of how novel high-fiber feed ingredients can improve poultry health and productivity.

## 5. Conclusions

Supplementing the diet of Zhedong white geese with SA does not significantly affect serum biochemical parameters or organ development. However, it has a positive effect on cecum length, cecal microbiota composition, and SCFA concentrations, though this is counterbalanced by a deterioration in intestinal morphology and nutrient digestibility in a high supplementation. Both antioxidant capacity and growth performance are enhanced with SA supplementation, but excessive levels lead to a decline. Importantly, this study highlights the innovative potential of using SA as a functional, high-fiber ingredient to optimize goose production and overall avian health. To translate these benefits effectively into practical poultry nutrition, we recommend maintaining an optimal supplementation level of 6% based on a comprehensive assessment of all influencing factors. Future research should explore the long-term effects of SA in different poultry species, investigate its molecular mechanisms of action, and assess potential synergies with other functional feed ingredients. Such studies could further reinforce the practical applicability of SA and pave the way for novel feeding strategies in commercial poultry systems.

## Figures and Tables

**Table 1:** The chemical composition of *Spartina anglica* (%, as-fed basis).

Crude Protein	Sodium Chloride	Cellulose	Hemicellulose	Lignin	Ash	Calcium	Phosphorus
5.66	1.75	33.15	27.13	24.62	1.50	0.53	0.10

**Table 2:** Composition and nutrient levels of diets (%, as-fed basis).

Items	0–42 Days	42–70 Days
Ingredients		
Corn	62.65	64.58
Soybean meal	22	15
Barley	10	15
Soybean oil	1	2
Calcium phosphate	2.2	1.6
Oyster shell powder	1	0.8
Salt	0.3	0.3
Lys-*L*, 98%	0.15	0.1
Met, 98%	0.2	0.12
Premix ^1^	0.5	0.5
Total	100	100
Analyzed nutrient levels		
Dry matter	87.1	86.5
Ether extract	5.35	5.1
Crude protein	16.06	13.79
Crude fiber	3.36	3.85
Neutral detergent fiber	12.87	13.16
Acid detergent fiber	3.78	4.24
Calculated nutrient levels ^2^		
ME, MJ/kg	12.13	12.55
Lys	1.01	0.85
Met	0.5	0.4
Thr	0.7	0.6
Calcium	0.85	0.75
Phosphorus	0.7	0.65

^1^ The premix provided the following per kg of diet: zinc (60 mg), iron (100 mg), manganese (80 mg), copper (10 mg), iodine (0.35 mg), selenium (0.3 mg), vitamin A (10,000 IU), vitamin D_3_ (2850 IU), vitamin E (30 IU), vitamin K_3_ (2 mg), vitamin B_12_ (1.2 mg), riboflavin (6 mg), nicotinic acid (40 mg), pantothenic acid (12 mg), pyridoxine (3 mg), biotin (0.2 mg), and choline chloride (800 mg). ^2^ These values were estimated based on the data reported by NRC 1994 [10].

**Table 3:** Effects of SA supplementation on growth performance in Zhedong white geese ^1^.

Items	Treatment				SEM	*p*-Value		
	CON	SA3	SA6	SA12		ANOVA	Linear	Quadratic
Initial body weight, g	115.08	113.90	114.82	115.95	0.81			
Final body weight, g	3156.5 ^b^	3401.14 ^a^	3429.86 ^a^	3075.5 ^b^	64.78	<0.01	0.13	<0.01
Average daily gain, g	43.45 ^b^	46.96 ^a^	47.36 ^a^	42.28 ^b^	0.92	<0.01	0.12	<0.01
Average daily feed intake, g	144.53 ^a^	150.8 ^a^	147.35 ^a^	138.88 ^b^	1.13	<0.01	<0.01	<0.01
FCR, g/g	3.35 ^b^	3.23 ^ab^	3.13 ^a^	3.31 ^b^	0.08	0.19	0.78	0.03

^a,b^ Values with different superscripts in the same row indicate significant differences at *p* < 0.05. SEM represents the pooled standard error of the means. ^1^ Each value represents the mean of six replicates.

**Table 4:** Effects of SA supplementation on nutrient digestibility of Zhedong white geese ^1^.

Items, %	Treatment				SEM	*p*-Value		
	CON	SA3	SA6	SA12		ANOVA	Linear	Quadratic
Dry matter	72.29	73.07	74.47	71.95	0.67	0.07	0.67	0.02
Organic matter	73.75	72.81	74.09	72.44	0.60	0.21	0.24	0.47
Crude protein	63.96 ^ab^	64.33 ^a^	65.32 ^a^	61.80 ^b^	0.57	<0.01	0.01	<0.01
Ether extract	86.77 ^a^	87.03 ^a^	88.94 ^a^	84.05 ^b^	0.55	<0.01	<0.01	<0.01

^a,b^ Values with different superscripts in the same row indicate significant differences at *p* < 0.05. SEM represents the pooled standard error of the means. ^1^ Each value represents the mean of six replicates.

**Table 5:** Effects of SA supplementation on serum biochemical parameters in Zhedong white geese ^1^.

Items	Treatment				SEM	*p*-Value		
	CON	SA3	SA6	SA12		ANOVA	Linear	Quadratic
ALT, IU/L	13.48	13.74	14.78	11.80	1.91	0.75	0.56	0.45
AST, IU/L	12.05	21.44	19.62	23.08	3.27	0.22	0.08	0.34
TP, g/L	44.70	46.84	45.65	43.03	1.71	0.50	0.37	0.30
ALB, g/L	14.58	13.74	14.57	13.82	0.48	0.54	0.51	0.95
GLOB, g/L	30.13	33.10	31.08	29.22	1.72	0.50	0.48	0.31
BUN, mmol/L	1.09	1.17	1.08	0.94	0.07	0.25	0.13	0.31
CHO, mmol/L	3.58	3.49	3.63	3.45	0.18	0.91	0.73	0.78
TG, mmol/L	0.96	1.39	0.94	1.15	0.21	0.49	0.89	0.83
HDL, mmol/L	1.73	1.75	1.52	1.76	0.21	0.84	0.98	0.55
LDL, mmol/L	1.52	1.41	1.73	1.4	0.08	0.08	0.6414	0.1501

^1^ Each value represents the mean of six replicates.

**Table 6:** Effects of SA supplementation on liver antioxidant capacity in Zhedong white geese ^1^.

Items	Treatment				SEM	*p*-Value		
	CON	SA3	SA6	SA12		ANOVA	Linear	Quadratic
SOD, U/mL	283.27 ^b^	341.66 ^a^	351.75 ^a^	357.91 ^a^	12.14	<0.01	<0.01	0.02
CAT, U/mL	10.67 ^b^	13.74 ^a^	16.15 ^a^	15.75 ^a^	0.62	<0.01	<0.01	<0.01
T-AOC, U/mL	7.87 ^b^	11.3 ^a^	12.98 ^a^	12.39 ^a^	0.55	<0.01	<0.01	<0.01
GSH-PX, U/mL	774.94 ^c^	907.64 ^bc^	1153.22 ^a^	1074.49 ^ab^	51.41	<0.01	<0.01	<0.01
MDA, nmol/mL	4.47	4.18	3.51	3.77	0.27	0.06	0.06	0.13

^a–c^ Values with different superscripts in the same row indicate significant differences at *p* < 0.05. SEM represents the pooled standard error of the means. ^1^ Each value represents the mean of six replicates.

**Table 7:** Effects of SA supplementation on serum antioxidant capacity in Zhedong white geese ^1^.

Items	Treatment				SEM	*p*-Value		
	CON	SA3	SA6	SA12		ANOVA	Linear	Quadratic
SOD, U/mL	106.66	110.19	114.68	113.87	2.51	0.17	0.10	0.54
CAT, U/mL	12.29	12.52	12.75	12.93	0.33	0.60	0.21	0.97
T-AOC, U/mL	8.92 ^b^	9.55 ^ab^	10.34 ^a^	10.01 ^ab^	0.27	0.02	0.03	0.14
GSH-PX, U/mL	753.68	791.02	846.80	829.73	19.83	0.06	0.07	0.42
MDA, nmol/mL	4.42	4.02	3.76	3.73	0.25	0.19	0.08	0.55

^a,b^ Values with different superscripts in the same row indicate significant differences at *p* < 0.05. SEM represents the pooled standard error of the means. ^1^ Each value represents the mean of six replicates.

**Table 8:** Effects of SA supplementation on organ indexes in Zhedong white geese ^1^.

Items	Treatment				SEM	*p*-Value		
	CON	SA3	SA6	SA12		ANOVA	Linear	Quadratic
Pancreatic	0.01	0.14	0.01	0.01	0.01	0.11	0.23	0.34
Kidney	0.81	0.82	0.86	0.80	0.06	0.88	0.95	0.98
Heart	0.65	0.82	0.74	0.77	0.04	0.06	0.14	0.08
Liver	2.38	2.86	2.31	2.52	0.16	0.11	0.34	0.17
Gallbladder	0.10	0.08	0.14	0.09	0.03	0.42	0.56	0.64
Spleen	0.13	0.14	0.15	0.16	0.02	0.91	0.65	0.98
Lungs	1.34	1.57	1.38	1.42	0.15	0.77	0.87	0.92
Uropygial gland	0.24	0.25	0.25	0.25	0.03	0.99	0.99	0.99
Thymus gland	0.24	0.22	0.25	0.23	0.04	0.95	0.89	0.96
Bursa of fabricius	0.05	0.08	0.08	0.07	0.07	0.27	0.32	0.45

^1^ Each value represents the mean of six replicates.

**Table 9:** Effects of SA supplementation on intestinal development in Zhedong white geese ^1^.

Items, cm	Treatment				SEM	*p*-Value		
	CON	SA3	SA6	SA12		ANOVA	Linear	Quadratic
Gizzard weight, % of BW	4.34	4.65	4.37	4.48	0.20	0.37	0.33	0.36
Duodenum length	38.75	35.90	38.02	38.05	2.32	0.91	0.42	0.81
Jejunum length	134.95	133.80	132.40	135.55	6.03	0.98	0.96	0.86
Ileum length	15.15	15.05	17.76	17.83	1.90	0.64	0.16	0.80
Cecum length	18.38 ^b^	18.50 ^b^	21.12 ^ab^	23.73 ^a^	0.99	0.01	0.03	0.24

^a,b^ Values with different superscripts in the same row indicate significant differences at *p* < 0.05. SEM represents the pooled standard error of the means. ^1^ Each value represents the mean of six replicates.

**Table 10:** Effects of SA supplementation on intestinal morphology in Zhedong white geese ^1^.

Items	Treatment				SEM	*p*-Value		
	CON	SA3	SA6	SA12		ANOVA	Linear	Quadratic
Jejunum								
Villus height, μm	876.41	884.39	904.65	753.76	45.79	0.18	0.07	0.21
Crypt depth, μm	308.66 ^ab^	281.28 ^b^	273.29 ^b^	346.95 ^a^	14.62	0.03	0.04	0.01
Villus height/crypt depth	2.84 ^a^	3.14 ^a^	3.33 ^a^	2.18 ^b^	0.13	<0.01	<0.01	<0.01
Ileum								
Villus height, μm	1109.48	1137.53	1294.66	1072.24	55.51	0.09	0.23	0.12
Crypt depth, μm	319.87 ^b^	331.97 ^b^	324.74 ^b^	388.17 ^a^	15.67	0.02	<0.01	0.22
Villus height/crypt depth	3.47 ^ab^	3.43 ^b^	3.97 ^a^	2.79 ^c^	0.11	<0.01	<0.01	<0.01

^a–c^ Values with different superscripts in the same row indicate significant differences at *p* < 0.05. SEM represents the pooled standard error of the means. ^1^ Each value represents the mean of six replicates.

**Table 11:** Effects of SA supplementation on cecum VFA concentrations of Zhedong white geese ^1^.

Items, mg/kg	Treatment				SEM	*p*-Value		
	CON	SA3	SA6	SA12		ANOVA	Linear	Quadratic
Acetic acid	911.15 ^c^	1241.05 ^bc^	1549.85 ^b^	2356.49 ^a^	163.12	<0.01	<0.01	0.66
Propanoic acid	347.54 ^b^	568.84 ^b^	648.45 ^ab^	1000.54 ^a^	85.71	<0.01	<0.01	0.98
Butyric acid	21.57 ^b^	46.73 ^a^	47.61 ^a^	47.4 ^a^	6.38	0.02	0.03	0.06
Valeric acid	20.45	24.64	20.49	27.99	4.81	0.75	0.42	0.80
Isovaleric acid	189.51 ^b^	461.63 ^b^	528.19 ^b^	921.66 ^a^	81.81	<0.01	<0.01	0.90

^a–c^ Values with different superscripts in the same row indicate significant differences at *p* < 0.05. SEM represents the pooled standard error of the means. ^1^ Each value represents the mean of six replicates.

## Data Availability

The original contributions presented in the study are included in the article, further inquiries can be directed to the corresponding authors.
